# Editorial: New insights in non-motor symptoms in Parkinson's disease

**DOI:** 10.3389/fneur.2024.1433934

**Published:** 2024-07-26

**Authors:** Cristian Falup-Pecurariu, Alessandra Fanciulli, Rupam Borgohain, Vinod Metta, K. Ray Chaudhuri

**Affiliations:** ^1^Department of Neurology, County Clinic Hospital Brasov, Brasov, Romania; ^2^Faculty of Medicine, Transilvania University Brasov, Brasov, Romania; ^3^Department of Neurology, Medical University of Innsbruck, Innsbruck, Austria; ^4^Parkinson's Disease and Movement Disorder Research Centre, Citi Neuro Centre, Hyderabad, India; ^5^Department of Basic and Clinical Neuroscience, Institute of Psychiatry, Psychology, and Neuroscience, King's College London, London, United Kingdom; ^6^Parkinson's Foundation Centre of Excellence, King's College Hospital, London, United Kingdom; ^7^Parkinson's Foundation Centre of Excellence, King's College Hospital London-Dubai, Dubai, United Arab Emirates

**Keywords:** Parkinson's disease, sleep disturbances, neuropsychiatric symptoms, pain, fatigue, gastrointestinal dysfunction, non-motor symptoms

In recent years, the wide range of non-motor symptoms encountered by Parkinson's disease (PD) patients have gained more attention of clinicians and researchers, as they have major impact on their quality of life. Key issues remain that of nonmotor fluctuations, pain, visual disturbances, sleep and other unmet needs. Establishing clinically meaningful biomarkers, screening questionnaires, characterizing the role of the microbiota-gut-brain axis, improving the concept of non-motor fluctuations, and the influence of device-aided therapies on non-motor symptoms are just some of the hot topics of research that contribute to a better understanding of non-motor symptoms of PD and their effective management (1–5).

In their original research, Diaconu et al., show the beneficial and sustained effects of levodopa-carbidopa intestinal gel (LCIG) therapy on sleep disturbances associated with PD. In this longitudinal study, 10 advanced PD patients were followed for 1 year after the initiation of LCIG therapy. They report improvement in several sleep parameters after LCIG therapy, suggesting that a proper management of the motor symptoms may also contribute to improved sleep quality.

In a brief research report, Marano et al., explore the importance of the vitamin D/parathormone (PTH) axis in the pathogenesis of restless legs syndrome (RLS) in PD. In their study of fifty PD patients, hyperparathyroidism was associated with the presence of RLS, independent of their motor disabilities due to PD. The authors postulate that identification and early treatment of such PD patients with vitamin D supplementation would reduce the levels of PTH and alleviate the symptoms of RLS.

Kurihara et al. used PainVision^®^, a perceptual pain/analyzer, to assess the pain threshold in PD patients. In their original research, the authors included 48 PD patients with pain and 53 PD patients without pain. Intensity of pain, as measured by PainVision^®^, did not correlate with traditional subjective rating scales, but PD duration and severity may be important factors that correlate with current pain perception threshold. The authors propose this new tool as an objective method for pain quantification in future research.

In an original research paper, Raeder et al. investigated the clinimetric properties of the Gut Dysmotility Questionnaire (GDQ), a newly developed assessment tool for screening and monitoring gastrointestinal dysfunction in PD, especially focusing on constipation. The Phase 1 of the research demonstrated the paucity of screening tools designed for the assessment of specific gastrointestinal symptoms in PD to date, therefore a preliminary version of the questionnaire was created. In Phase 2, cognitive pretests (such as standard pretests, interviews and assessment questionnaires) were conducted to develop the final version of the GDQ, which comprises of 18 items for the self-assessment of the gastrointestinal dysmotility. GDQ showed high acceptance and efficacy, as well as sufficient reliability and construct validity in the present study, and it may become a promising assessment tool of the gastrointestinal symptoms in future clinical practice and research.

Although motor fluctuations are known to negatively influence the quality of life of the PD patients, less is known about the non-motor fluctuations. In their original research article, Kakimoto et al., explored the role of non-motor fluctuations in relation to motor fluctuations and quality of life. Non-motor fluctuations were found in 26.1% of the PD patients enrolled in the study and were observed even in early stages of the disease. Non-motor fluctuations were shown to impair the quality of life of PD patients, independently of motor fluctuations, suggesting that their early identification is key for an optimized therapeutic outcome.

The evolution of dysautonomia in PD was observed in a longitudinal study conducted by Stewart et al.. For this purpose, information from the Parkinson's Progression Markers Initiative (PPMI) database was assessed. Autonomic dysfunction (evaluated by several specific scales) was observed since the early stages of the disease and worsened over the 7 years of follow-up. The positive association between greater olfactory dysfunction, more autonomic impairment, and more severe motor symptoms with respect to baseline may offer new insights regarding the pathological processes underlying PD progression.

Jiang et al., evaluated the main characteristics of sleep using polysomnography and questionnaires in 44 naïve PD patients. Nocturnal awakenings and reduced sleep efficiency were the most common features of poor sleep quality in these PD patients. More than half of the subjects had a poor sleep quality, which was associated with the severity of non-motor symptoms and a reduced quality of life. Sleep may also influence the evolution of motor symptoms, as the authors observed that an increased number of nocturnal arousals events may predict the progression of motor impairment.

The pathogenesis of the neurodegenerative processes underlying the development of PD are still not fully understood. Li et al. highlighted in their review the complex role of the microbiota-gut-brain axis in the pathophysiology of PD. The authors cover several aspects that may explain the mechanisms of neurodegeneration in PD (the body-first and brain-first theories, the role of the microbiome dysregulation, the changes in metabolites derived from gut microbiome, the influence of the genetic and environmental factors, etc.), taking also into consideration potential targets for future management or prevention plans.

Zhao et al. conducted a meta-analysis to evaluate the role of white matter hyperintensities (WMH), on cognitive function in PD. Pooling results from 23 studies, they showed that WMH an imaging marker of the white matter in disease causation, was associated with cognitive impairment in PD.

In a review article, Ungureanu et al., describe dry eye disease in PD. With an increased estimated prevalence among 70% of PD patients, dry eyes can manifest through a large burden of symptoms, such as excessive tearing, visual discomfort/disturbances and photophobia, that impair patients' quality of life. The authors focus on the pathogenesis, clinical evaluation, and management of this non-motor symptom commonly encountered in PD.

Pain is another non-motor symptom with a high prevalence in PD (up to 85%), complex interactions with motor symptoms, other non-motor features and quality of life. Alizadeh et al., explored in a review paper the potential links between several genotypes and pain symptoms in monogenic PD. A good understanding of the genetic profile in patients with specific types of pain may contribute to tailored therapeutic approaches for patients with monogenic PD in the future.

Research on non-motor symptoms in PD is much needed for a better understanding of the pathophysiology of this disease and improving the care of PD patients.

## Author contributions

CF-P: Writing – original draft, Writing – review & editing. AF: Writing – review & editing. RB: Writing – review & editing. VM: Writing – review & editing. KC: Writing – review & editing.
